# Beyond Gallstones and Alcohol: Unveiling Hypertriglyceridemia as the Cause of Pancreatitis

**DOI:** 10.7759/cureus.106536

**Published:** 2026-04-06

**Authors:** G K M Rashik Uzzaman, Monowara Rahman Shipi, Nusrat Jahan, Manoj Kumar Mahadevaswamy Susheela, Cornelius Fernandez

**Affiliations:** 1 General Medicine, United Lincolnshire Teaching Hospitals NHS Trust, Lincoln, GBR; 2 Acute Medicine, United Lincolnshire Teaching Hospitals NHS Trust, Boston, GBR; 3 Internal Medicine, United Lincolnshire Teaching Hospitals NHS Trust, Boston, GBR; 4 Diabetes and Endocrinology, United Lincolnshire Teaching Hospitals NHS Trust, Boston, GBR; 5 Endocrinology, United Lincolnshire Teaching Hospitals NHS Trust, Lincoln, GBR

**Keywords:** alcohol use, intensive care unit stay, severe acute pancreatitis, severe hypertriglyceridemia, severe pancreatitis

## Abstract

Hypertriglyceridemia is a less frequent yet clinically significant cause of acute pancreatitis, accounting for approximately 4-10% of cases, and is often overlooked when routine laboratory analysis is hindered by lipemia-related analytical interference. We describe two cases of severe hypertriglyceridemia-induced pancreatitis presenting with acute abdominal pain and markedly elevated triglyceride levels. The first case involved polygenic hypertriglyceridemia triggered by undiagnosed diabetes. The second case represented secondary, alcohol-related hypertriglyceridemia. In both cases, lipemia-related laboratory interference complicated the initial evaluation, but prompt recognition and treatment with intravenous insulin led to rapid clinical and biochemical improvement. These cases highlight the need for early consideration of hypertriglyceridemia in pancreatitis of unclear etiology, awareness of laboratory artifacts, and prompt triglyceride-lowering therapy to prevent complications.

## Introduction

Acute pancreatitis is a common yet multifaceted clinical entity, with biliary pathology and alcohol misuse being the most frequent causes. Hypertriglyceridemia is a less common but clinically significant cause of acute pancreatitis, accounting for approximately 4-10% of cases, and may be overlooked, particularly when laboratory evaluation is affected by lipemia-related analytical interference [1]. Severe hypertriglyceridemia can precipitate pancreatitis through free fatty acid-mediated pancreatic injury and increased blood viscosity, leading to pancreatic ischemia. We report two cases of severe hypertriglyceridemia-induced pancreatitis with distinct underlying mechanisms - one genetic and one acquired - to illustrate diagnostic challenges and management considerations. This study explores a diagnostically elusive presentation of pancreatitis where classical indicators were misleading, and the key lay hidden beneath a layer of lipemic serum. Through methodical exclusion and biochemical insight, hypertriglyceridemia was revealed as the true culprit, underscoring the importance of clinical suspicion and systematic evaluation in atypical presentations.

## Case presentation

Case 1

A 28-year-old gentleman presented to the emergency department with acute abdominal pain associated with vomiting. The pain was acute in onset, constant, and severe in intensity (9/10), predominantly located in the epigastrium with radiation to the back. It was non-colicky in nature, associated with nausea and multiple episodes of vomiting, aggravated by movement, and partially relieved by leaning forward.

He was previously fit and well with no significant past medical history and was not taking any regular medications, apart from a topical cream recently prescribed by his general practitioner for generalized pruritic skin lesions, which had not provided relief. He did not smoke or consume alcohol but reported drinking up to five cans of energy drinks daily over the preceding year. There was no significant family history. He lived with his wife and worked in the vegetable packaging industry.

On examination, he was morbidly obese with a BMI of 41.6 kg/m² (height 180 cm, weight 135 kg). On arrival at the emergency department, he was mildly tachycardic and tachypneic, with otherwise stable hemodynamics. His observations included a pulse rate of 101/minute, a respiratory rate of 24/minute, a blood pressure of 143/72 mmHg, a temperature of 37.7°C, and an oxygen saturation of 96% on 2 L of oxygen. Cardiovascular and respiratory examinations were unremarkable. Abdominal examination revealed a soft abdomen with tenderness in the epigastrium and right hypochondrium, and bowel sounds were normal.

**Figure 1:**
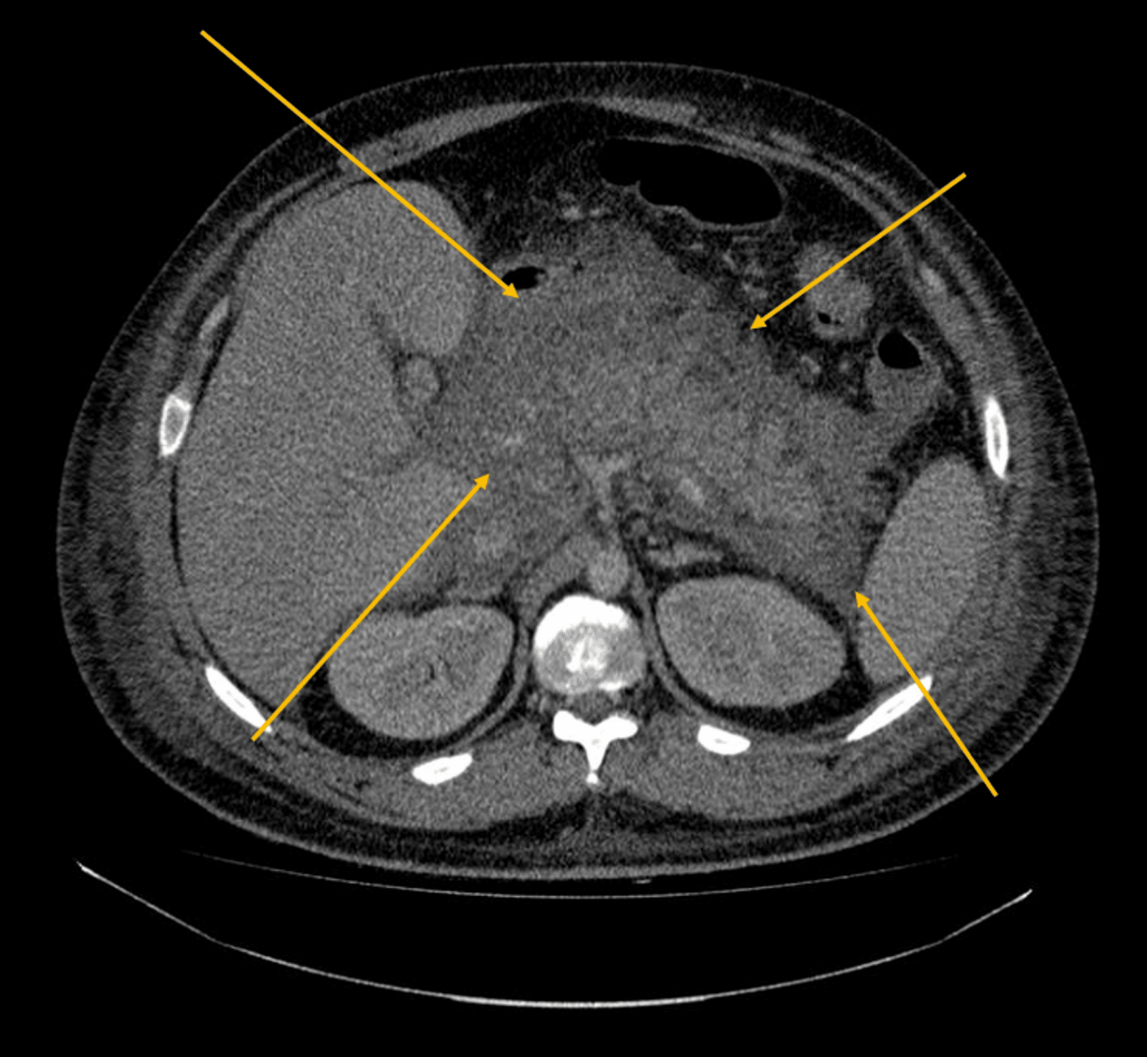
CT scan of the abdomen of Case 1 showed evidence of acute pancreatitis without focal necrosis. The arrows highlight radiological features consistent with acute pancreatitis, including pancreatic enlargement and surrounding peripancreatic inflammatory changes.

Initial venous blood gas analysis showed hyperglycemia with borderline ketone elevation but no significant acidosis (Table 1). He was started on intravenous fluids and analgesia, and urinary catheterization was performed to monitor urine output. A CT scan of the abdomen demonstrated features of acute pancreatitis without focal necrosis, gallstones, or common bile duct dilatation (CT severity index 3-4/10) (Figure 1). The surgical team recommended conservative management.

**Table 1:** Laboratory findings for patient of Case 1. HDL: high-density lipoprotein; TC: total cholesterol; GFR: glomerular filtration rate; CRP: C-reactive protein; WBC: white blood cell; HbA1c: glycated hemoglobin; TSH: thyroid stimulating hormone; FT4: free T4

Parameters	Patient value	Reference range
pH	7.42	7.35-7.45
Bicarbonate	19.7 mmol/L	22-28 mmol/L
Base excess	-3.38 mmol/L	-2 to +2 mmol/L
Lactate	1.9 mmol/L	1-2.5 mmol/L
Glucose	18.9 mmol/L	3-6 mmol/L
Capillary ketones	2.1 mmol/L	<0.6 mmol/L
Triglycerides	145.6 mmol/L	<1.7 mmol/L
Total cholesterol	28.6 mmol/L	<5.0 mmol/L
HDL cholesterol	0.4 mmol/L	>1.0 mmol/L (men)
Non-HDL cholesterol	28.2 mmol/L	<4.0 mmol/L
TC/HDL ratio	71.5	<4.5
Potassium	4.6 mmol/L	3.5-5.5 mmol/L
Urea	3.3 mmol/L	2.5-7.8 mmol/L
Creatinine	98 µmol/L	59-104 µmol/L
GFR	>90 mL/min	90-200 mL/min
Bilirubin	23 µmol/L	0-21 µmol/L
Alkaline phosphatase	60 U/L	30-130 U/L
Albumin	24 g/L	35-50 g/L
Calcium (adjusted)	1.73 mmol/L	2.2-2.6 mmol/L
Phosphate	0.21 mmol/L	0.8-1.5 mmol/L
CRP	196 mg/L	0-5 mg/L
Hemoglobin	158 g/L	130-170 g/L
WBC	13.8 ×10⁹/L	4.3-11.2 ×10⁹/L
Platelets	212 ×10⁹/L	150-400 ×10⁹/L
Neutrophils	11.31 ×10⁹/L	2.1-7.4 ×10⁹/L
Amylase	151 U/L	28-100 U/L
HbA1c	113 mmol/mol	20-41 mmol/mol
TSH	1.7 mU/L	0.27-4.5 mU/L
FT4	11.1 pmol/L	11-23 pmol/L
Apolipoprotein B	172 mg/dL	66-133 mg/dL
Apolipoprotein A1	71 mg/dL	104-202 mg/dL

A few hours after admission, his clinical condition deteriorated, with worsening tachycardia and tachypnea. Repeat venous blood gas analysis demonstrated metabolic acidosis with rising glucose levels and increased ketones. He was subsequently diagnosed with diabetic ketoacidosis (DKA), likely secondary to acute pancreatitis, and was started on the DKA management protocol.

Multiple blood samples sent for laboratory analysis were reported as markedly lipemic, preventing measurement of routine parameters, including urea and electrolytes (U&E), full blood count (FBC), CRP, and amylase. The available lipid profile demonstrated severe hypertriglyceridemia, with triglycerides of 145.6 mmol/L (12,890 mg/dL), total cholesterol of 28.6 mmol/L (1105 mg/dL), and HDL cholesterol of 0.4 mmol/L (15 mg/dL) (Table 1).

A repeat sample obtained a few hours after initiation of the DKA protocol showed elevated inflammatory markers with preserved renal function (Table 1). However, sodium, alanine aminotransferase (ALT), and amylase remained unmeasurable due to persistent lipemia-related analytical interference. The first measurable amylase level, obtained 24 h after admission, was mildly elevated.

At this stage, we could calculate the Glasgow-Imrie criteria for the severity of acute pancreatitis, and he scored 4, indicating a high risk for severe pancreatitis [1]. With this combination of high risk of severe pancreatitis and diabetic ketoacidosis, the patient was taken to the intensive care unit (ICU) and was managed conservatively with in-vitro fertilization (IVF) under hemodynamic monitoring, DKA protocol, patient-controlled analgesia, and nasogastric (NG) feeding.

In the intensive care unit, the endocrinology team reviewed the patient and raised the possibility of severe hypertriglyceridemia as the primary event precipitating acute pancreatitis, which subsequently triggered diabetic ketoacidosis. Examination revealed multiple yellowish papular lesions over the buttocks, back, and extensor surfaces of the extremities, consistent with eruptive xanthomas. There was no evidence of lipemia retinalis or hepatosplenomegaly. Laboratory investigations showed markedly elevated HbA1c, suggesting pre-existing diabetes mellitus, while thyroid function tests were within normal limits (Table 1). The endocrinology team recommended continuation of variable-rate intravenous insulin infusion (VRIII) even after resolution of DKA until triglyceride levels approached near-normal values.

CT scan repeated three days after admission showed no evidence of pancreatic necrosis. However, there was evidence of significant peripancreatic inflammation, with ascites and bilateral pleural effusion. His lipid profile markedly improved with intravenous insulin treatment. The lipidologist’s opinion was sought, who suggested that the most likely reason for his acute pancreatitis is chylomicronemia syndrome. Blood samples were sent to a reference laboratory for the lipoprotein electrophoresis and molecular genetic studies. A strict low-fat diet was initiated, and he was started on fenofibrate 160 mg and atorvastatin 40 mg once daily.

On the ninth day of admission, he was pain-free; his amylase had dropped to 15 U/L; his lipid profile had improved (total cholesterol 4.7 mmol/L, TG 3.8 mmol/L, HDL cholesterol 0.4 mmol/L, LDL cholesterol 2.56 mmol/L, non-HDL cholesterol 4.3 mmol/L); and his blood glucose levels were stable on a basal-bolus regimen. He was stepped down to the ward and discharged in a few days with advice regarding risk of recurrent pancreatitis, the need for strict adherence to a low-fat diet (<15% of calories as fat), optimal diabetic control, weight reduction, and avoidance of alcohol and energy drinks. By the time of discharge, eruptive xanthomas had almost disappeared.

These results arrived a few weeks after discharge. Islet antibodies (GAD65, ZnT8, and IA-2) were negative, suggesting non-autoimmune diabetes mellitus. Given the patient's morbid obesity (BMI 41.6 kg/m²), severe insulin resistance, and markedly elevated HbA1c (113 mmol/mol), the diabetes was considered most consistent with newly diagnosed type 2 diabetes mellitus (ApoB: 172 {66-133} mg/dL, ApoA1: 71 {104-202} mg/dL). The absence of pathogenic variants in lipoprotein lipase (LPL) and related genes supported a diagnosis of polygenic hypertriglyceridemia (Fredrickson type IIb), exacerbated by diabetes and high-energy drink intake. Molecular genetics did not show evidence of mutation in the genes encoding lipoprotein lipase (LPL) or its cofactors, including Apolipoprotein C2 (APOC2), Apolipoprotein A5 (APOA5), lipase maturation factor 1 (LMF1), glycosylphosphatidylinositol-anchored high-density lipoprotein-binding protein 1 (GPIHBP1), and glycerol-3-phosphate dehydrogenase 1 (GPD1). Unfortunately, he lost to follow-up.

Case 2

A 44-year-old male presented to the emergency department with acute epigastric pain for 6 h, which was associated with one episode of vomiting. He described the pain as 9/10 on the pain scale, which was exacerbated on inspiration and was non-radiating. He was fit and well with no significant past medical history other than fatty changes in the liver. He is not on any medications. He was drinking around 150 mL of vodka every day for the last six years and smoking five cigarettes every day for the last 20 years. He does not have any significant family history apart from his father, who died due to a heart attack. He is of Latvian origin and lives with his partner and works in a factory.

On arrival to the emergency department, his observations were as follows: pulse rate 127 beats/minute, respiratory rate 18 breaths/minute, blood pressure 136/99 mmHg, temperature 36.4°C, and oxygen saturation 99% on room air. His respiratory and cardiovascular examinations were normal. His abdomen was soft, with severe epigastric tenderness without muscle guarding. Bowel sounds were present. His venous blood gas on arrival showed a pH of 7.359 (7.35-7.45), bicarbonate of 27.8 (22-28) mmol/L, base excess of -1 (-2 to +2) mmol/L, lactate 1.4 (1-2.5) mmol/L, and glucose of 5.6 (3-6) mmol/L. His capillary blood ketones were 1 mmol/L (Table 2).

**Table 2:** Laboratory findings of Case 2.

Parameters	Patient value	Reference range
pH	7.359	7.35-7.45
Bicarbonate	27.8 mmol/L	22-28 mmol/L
Base excess	-1 mmol/L	-2 to +2 mmol/L
Lactate	1.4 mmol/L	1-2.5 mmol/L
Glucose	5.6 mmol/L	3-6 mmol/L
Capillary ketones	1 mmol/L	<0.6 mmol/L
Triglycerides	79.8 mmol/L	<1.7 mmol/L
Total cholesterol	42.6 mmol/L	<5.0 mmol/L

**Figure 2:**
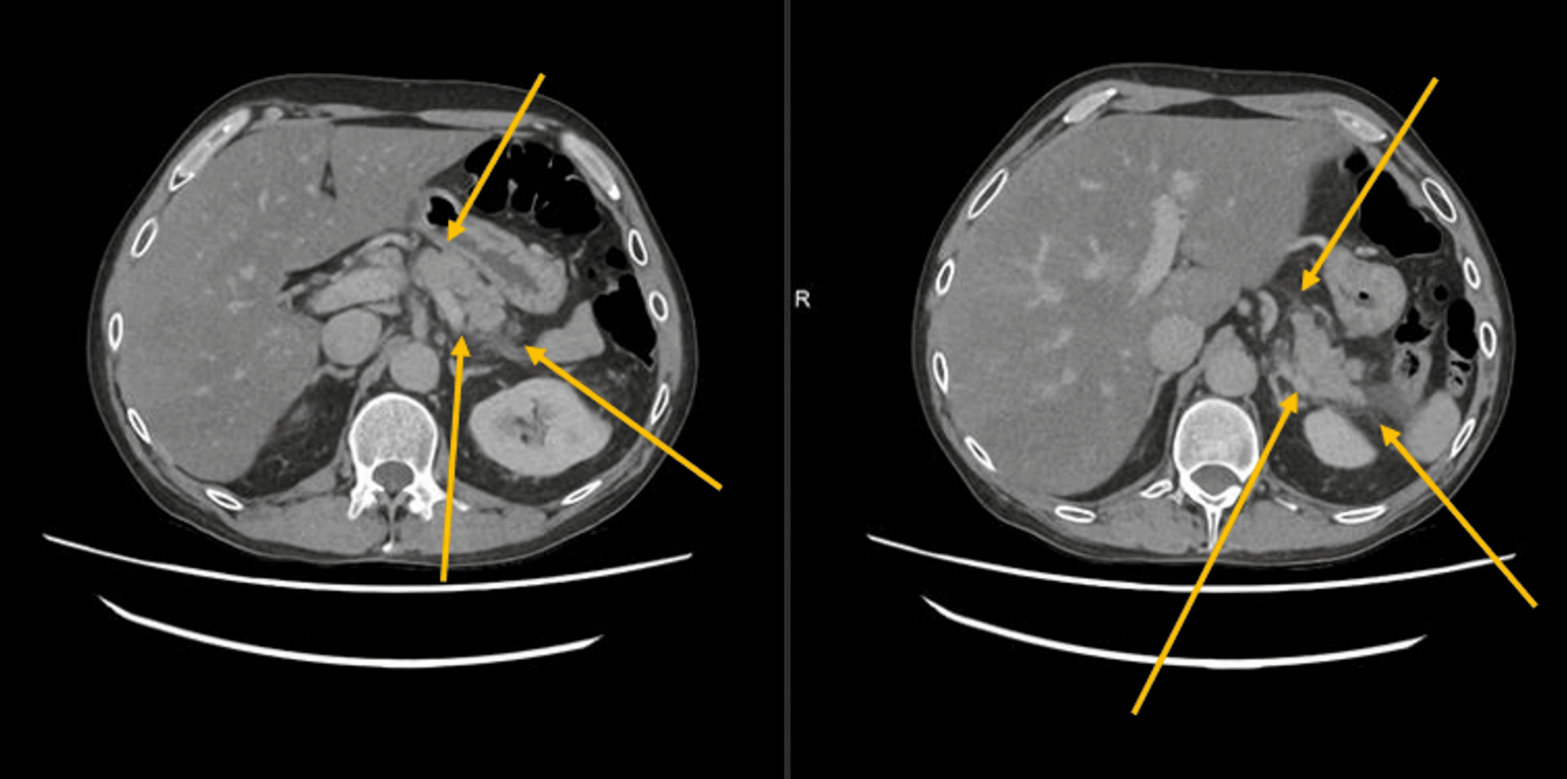
CT scan of the abdomen of Case 2 revealed features suggestive of acute pancreatitis without pancreatic necrosis. The arrows highlight radiological features consistent with acute pancreatitis, including pancreatic enlargement and surrounding peripancreatic inflammatory changes.

CT scan of the abdomen revealed features suggestive of acute pancreatitis and liver steatosis with no obvious evidence of pancreatic necrosis (Figure 2). An ultrasound of the abdomen was subsequently performed to evaluate for biliary etiology, particularly gallstones or biliary obstruction, which are common causes of acute pancreatitis. The ultrasound revealed a mildly hyperechoic liver in keeping with fatty changes, with no evidence of gallstones or biliary dilatation, and normal hepatoportal blood flow within the portal vein. The surgical team advised conservative management.

Multiple blood samples were reported as lipemic, preventing analysis of U&E, CRP, clotting profile, and amylase. The laboratory could give us only the cholesterol and TG, which were as follows: total cholesterol 42.6 mmol/L and, most importantly, TG 79.8 mmol/L (Table 2). The patient was managed conservatively with IVF, patient-controlled analgesics, and VRIII in order to manage the hypertriglyceridemia. The patient also received vitamin B complex with ascorbic acid supplementation, given the history of chronic alcohol consumption. He was also started on atorvastatin 80 mg and thiamine. Following treatment with VRIII, his lipid profile improved dramatically, and TG returned to normal on the third day. However, the total cholesterol level was on the higher side (25.4 mmol/L). The rapid biochemical normalization with insulin infusion confirmed the diagnosis of secondary hypertriglyceridemia responsive to metabolic correction.

The patient was completely pain-free on the fourth day of admission, the lipid level improved, and the blood glucose was stable. He was discharged with advice regarding the risk of recurrent pancreatitis, the need for strict adherence to a low-fat diet (<15% of calories as fat), and avoidance of alcohol and energy drinks. Ophthalmology evaluation in the outpatient setting has not revealed any evidence of lipemia retinalis. Molecular genetics did not show evidence of mutation in the genes known to cause hypertriglyceridemia, depicting this as acquired hypertriglyceridemia secondary to excessive alcohol intake.

## Discussion

These case reports juxtapose two clinically similar yet etiologically distinct presentations of hypertriglyceridemia-induced acute pancreatitis (HTG-AP). While both patients presented with acute abdominal pain, lipemic serum, and extremely elevated TG levels, the underlying causes were fundamentally different - one rooted in primary (genetic) hyperlipoproteinemia with a contribution from two environmental triggers (undiagnosed diabetes and excessive consumption of energy drinks), and the other was purely caused by an environmental trigger (excessive alcohol consumption).

Mechanism of hypertriglyceridemia-associated acute pancreatitis

Nearly 5-10% of all cases of acute pancreatitis are due to hypertriglyceridemia, whereas gallstone disease and alcohol cause 40% and 30% of acute pancreatitis [1]. As per Endocrine Society guidelines, TG levels in the severe hypertriglyceridemia range (1.29-22.58 mmol/L or 1000-1999 mg/dL) and in the very severe hypertriglyceridemia range (>22.59 mmol/L or 2000 mg/dL) carry a high risk for acute pancreatitis by 5% and 10-20%, respectively [2]. It is observed that the incidence of acute pancreatitis increases by 3% for every 1.1 mmol/L rise in TG levels over 11.29 mmol/L [3].

The mechanism for HTG-AP is a free radical damage initiated by the free fatty acids (FFAs) that are generated by the pancreatic lipase acting on TG. Another mechanism is hypertriglyceridemia-induced hyperviscosity leading to pancreatic ischemia and inﬂammation. Classically, hypertriglyceridemia-induced acute pancreatitis (HTG-AP) is severe, recurrent, and life-threatening, often presenting as necrotizing (in nearly 50%) or hemorrhagic pancreatitis [4]. In our cases, although pancreatic necrosis was not observed, both patients exhibited severe biochemical derangements and systemic involvement, underscoring the potential severity of HTG-AP even in the absence of necrosis. After a few acute episodes, patients might develop chronic pancreatitis with exocrine or endocrine pancreatic insufficiency, including pancreatic diabetes mellitus [4]. Moderate hypertriglyceridemia can occur during acute pancreatitis [5]. Thus, moderate hypertriglyceridemia during an acute pancreatitis should not be considered causal.

Bidirectional relationship between DKA and acute pancreatitis

Acute pancreatitis can coexist with DKA in 10-15% of cases [5]. Acute pancreatitis can cause DKA due to insulin deficiency caused by the pancreatic parenchymal injury and counter-regulatory hormone excess caused by the systemic inflammatory response associated with acute pancreatitis [5]. In a reciprocal relationship, DKA can cause acute pancreatitis. DKA is associated with moderate hypertriglyceridemia and is mediated by insulin deficiency, which increases lipolysis and FFA delivery to the liver, coupled with inhibition of lipoprotein lipase in peripheral tissues, leading to increased hepatic very low-density lipoprotein (VLDL) production. The accumulated TG is toxic to the pancreas, triggering acute pancreatitis. Moderate hypertriglyceridemia that is frequent during DKA usually gets normalized within 24-72 h of insulin therapy [6]. However, severe hypertriglyceridemia that develops during DKA can result in acute pancreatitis. Acute pancreatitis associated with DKA is generally a mild disease [5]. This is in contrast to the acute pancreatitis that develops with severe hypertriglyceridemia, in which case the pancreatitis is necrotizing or hemorrhagic.

Causes of primary and secondary hypertriglyceridemia

Primary (Genetic) - Fredrickson Classification

These are usually familial disorders of lipid metabolism due to mutations affecting enzymes/proteins involved in TG clearance (Table 3) [7].

**Table 3:** Fredrickson classification of hyperlipoproteinemia and associated lipoprotein abnormalities. VLDL: very low-density lipoprotein; LDL: low-density lipoprotein; IDL: intermediate-density lipoprotein; LPL: lipoprotein lipase

Types	Disorders	Lipoprotein abnormality	Triglyceride level	Mechanism
I	Familial chylomicronemia	↑ Chylomicrons	Very high	LPL or ApoC2 deficiency
IIb	Combined hyperlipidemia	↑ LDL, ↑ VLDL	Moderate to high	Increased ApoB production
III	Dysbetalipoproteinemia	↑ IDL	Moderate to high	ApoE2 homozygosity → remnant accumulation
IV	Familial hypertriglyceridemia	↑ VLDL	Moderate to high	Increased VLDL production
V	Mixed hyperlipidemia	↑ VLDL and ↑ chylomicrons	Very high	Combined VLDL overproduction and impaired clearance

Secondary (Acquired) Causes

These causes are external factors that lead to elevated serum TG levels (Table 4) [2]. Secondary hypertriglyceridemia is often multifactorial and may result from metabolic conditions, endocrine disorders, lifestyle factors, medications, or systemic illnesses. Identifying these reversible contributors is essential, as management of the underlying condition can significantly reduce triglyceride levels and prevent complications.

**Table 4:** Secondary causes of hypertriglyceridemia. FFA: free fatty acid; VLDL: very low-density lipoprotein; TG: triglycerides; LPL: lipoprotein lipase; HAART: highly active antiretroviral therapy This table has been adapted from Berglund et al. [2], which is an open-access article distributed under the terms and conditions of the Creative Commons CC-BY-NC-ND license.

Causes	Mechanisms
Uncontrolled diabetes mellitus	Insulin deficiency → ↑ lipolysis and VLDL production
Obesity/metabolic syndrome	Insulin resistance → ↑ FFA → ↑ hepatic TG synthesis
Excessive alcohol intake	Stimulates VLDL production, inhibits LPL
Hypothyroidism	Reduces clearance of lipoproteins
Chronic kidney disease	Altered lipoprotein metabolism
Nephrotic syndrome	Hepatic overproduction of lipoproteins
Pregnancy	Estrogen → ↑ hepatic VLDL synthesis
Medications	Thiazides, beta-blockers, corticosteroids, estrogen, isotretinoin, antipsychotics, protease inhibitors
High-fat/high-sugar diet	Provides substrate for excess triglyceride synthesis
Acute pancreatitis	May result from or contribute to severe hypertriglyceridemia
HIV/HAART therapy	Protease inhibitors cause lipid abnormalities

Severity of Hypertriglyceridemia

According to the 2012 Endocrine Society Clinical Practice Guidelines on the Evaluation and Treatment of Hypertriglyceridemia, the severity of hypertriglyceridemia is classified based on fasting TG levels as presented in Table 5 [2].

**Table 5:** Severity of hypertriglyceridemia. This table has been adapted from Berglund et al. [2], which is an open-access article distributed under the terms and conditions of the Creative Commons CC-BY-NC-ND license.

Severity	Triglyceride level (mg/dL)	Triglyceride level (mmol/L)
Normal	<150	<1.7
Mild	150-199	1.7-2.2
Moderate	200-999	2.3-11.2
Severe	1000-1999	11.3-22.5
Very severe	≥2000	≥22.6

Molecular genetics of severe hypertriglyceridemia

Severe hypertriglyceridemia, as seen in Case 1, was historically considered a monogenic disorder caused by mutations affecting genes involved in chylomicron catabolism, including lipoprotein lipase (LPL), apolipoprotein C2 (APOC2), apolipoprotein A5 (APOA5), lipase maturation factor 1 (LMF1), glycosylphosphatidylinositol-anchored high-density lipoprotein-binding protein 1 (GPIHBP1), and glycerol-3-phosphate dehydrogenase 1 (GPD1). These mutations typically follow an autosomal recessive inheritance pattern and are rare, with an estimated prevalence of 1-10 per million [8,9]. Individuals who are homozygous or compound heterozygous for these mutations usually develop severe hypertriglyceridemia, whereas heterozygous carriers may develop moderate hypertriglyceridemia [8].

However, monogenic hypertriglyceridemia is uncommon, and most patients with severe hypertriglyceridemia have a polygenic form in which multiple genetic susceptibilities interact with environmental factors to precipitate disease [9]. The absence of pathogenic monogenic variants in our first patient supports the increasingly recognized predominance of polygenic hypertriglyceridemia, likely exacerbated by uncontrolled diabetes and excessive energy drink consumption.

Molecular genetics of moderate hypertriglyceridemia

Moderate hypertriglyceridemia is commonly polygenic in inheritance and is caused by mutations in multiple genes that influence VLDL production and clearance. The hyperlipoproteinemia types including IIb, III, IV and V are polygenic [10]. Though they look different in biochemical phenotype, they closely resemble each other at the genetic level [10]. Hence, all hyperlipoproteinemia subtypes except type I and IIa should be considered as a single group, and hence lipoprotein electrophoresis is of less importance when compared to molecular genetics [10].

Laboratory artifacts in DKA and severe hypertriglyceridemia

Diabetic ketoacidosis (DKA) can cause asymptomatic elevations of pancreatic amylase and lipase in 16-25% of cases [11]. Therefore, in patients with DKA, acute pancreatitis should not be diagnosed solely on the basis of elevated pancreatic enzymes, even when levels exceed three times the upper limit of normal [11]. Conversely, pancreatic enzymes may be falsely normal in up to 50% of hypertriglyceridemia-induced acute pancreatitis due to assay interference, potentially delaying diagnosis [12]. Thus, the diagnosis of acute pancreatitis in the setting of DKA and hypertriglyceridemia should rely on clinical assessment and imaging studies.

Extreme hypertriglyceridemia may also interfere with laboratory measurements, resulting in pseudohyponatremia and pseudonormoglycemia. The latter occurs because lipemic serum reduces the aqueous phase of plasma, leading to spuriously low glucose measurements despite true hyperglycemia, which may mask DKA and present as apparent euglycemic DKA. Repeated testing with serial dilution can help overcome these interferences caused by hyperlipidemic serum [11]. Similarly, assay interference may result in spuriously elevated transaminases, making it difficult to exclude coexistent alcoholic liver disease.

Comparative overview: primary vs secondary hypertriglyceridemia

Table 6 summarizes the key differences between the two cases. Together, these cases underscore the multifactorial nature of hypertriglyceridemia-induced pancreatitis and the value of integrating clinical, biochemical, and genetic data for accurate etiologic classification.

**Table 6:** Table showing the major differences between Case 1 and Case 2. HTG: hypertriglyceridemia; VRIII: variable-rate intravenous insulin infusion

Feature	Case 1 (primary HTG)	Case 2 (secondary HTG)
Age	28 years	44 years
Comorbidities	Newly diagnosed diabetes with obesity	Alcohol use disorder with fatty liver disease
Precipitating factor	Undiagnosed diabetes and energy drinks	Chronic alcohol intake
Triglyceride peak	145.6 mmol/L	79.8 mmol/L
Genetic testing	Type IIb phenotype, no monogenic mutations	No mutation
Dermatological features	Eruptive xanthomas	Absent
Management approach	Insulin, VRIII, statin, fibrate	Insulin, statin, vitamin B-complex
Follow-up outcome	Lost to follow-up	Discharged with lipid control advice

## Conclusions

These cases illustrate that both polygenic and acquired forms of hypertriglyceridemia can precipitate severe pancreatitis, underscoring the importance of early recognition and targeted management. A tailored approach based on genetic risk, lifestyle triggers, and metabolic context is essential for optimal outcomes and prevention of recurrence.
